# Intranasal lithium in Ryanodex formulation vehicle inhibits inflammation and pyroptosis in aged 5XFAD mice brains

**DOI:** 10.1002/alz.089654

**Published:** 2025-01-09

**Authors:** Piplu Bhuiyan, Wenjia Zhang, Rebecca Chae, Kyelee Kim, Lauren St. Louis, Ying Wang, Ge Liang, Jia Liu, Huafeng Wei

**Affiliations:** ^1^ Department of Anesthesiology and Critical Care, Perelman School of Medicine, University of Pennsylvania, Philadelphia, PA 19104, U.S.A., Philadelphia, PA USA

## Abstract

**Background:**

This study investigates the therapeutic versus side effects of intranasal lithium chloride (LiCl) in Ryanodex formulation vehicle (RFV) to inhibit inflammation and pyroptosis and to ameliorate on cognitive dysfunction and depressive behavior in 5XFAD mice.

**Method:**

5XFAD and wild type (WT) B6SJLF1/J mice were treated with intranasal or oral LiCl (3 mM/kg) dissolved in RFV starting at 2 or 9 months old and the continuous treatment lasted for 12 weeks. Behavior was examined for depression, cognition, olfaction, and motor function at the ages of 5 or 12 months. Body weights were monitored monthly. At 6 or 13 months old, blood and brain were harvested. Blood biomarkers for thyroid (TSH: thyroid stimulating hormone) and kidney (creatinine) function were measured by ELISA. Immunoblotting determined the proteins expression levels of type 1 InsP_3_ receptors (InsP_3_R‐1), oxidative stress (4‐HNE, MDA), pyroptosis activation pathway regulatory proteins, proinflammatory versus neuroprotective cytokines, and synaptic proteins in aged mice brains.

**Result:**

Compared to WT mice, significant memory loss and depression behavior existed in both adult and aged 5XFAD mice, and both were abolished by intranasal LiCl in RFV Intranasal LiCl in RFV significantly inhibited the pathological proteins increase of InsP_3_R‐1, MDA‐modified proteins by pyroptosis pathway activation proteins (NLRP3, cleaved caspase‐1, N‐terminal GSDMD) in aged 5XFAD mice brains, in comparison with aged WT mice brains. Furthermore, intranasal LiCl in RFV significantly reduced protein levels of cytotoxic cytokines (IL‐1β, IL‐18, IL‐6, TNF‐α), alleviated the activation of astrogliosis and microgliosis, and increased cytoprotective cytokine IL‐10 and inhibited PSD‐95 synapse protein loss in aged 5XFAD mice brain. Intranasal LiCl did not affect blood TSH but significantly inhibited abnormal age‐dependent increase of blood creatinine in 5XFAD mice. Intranasal LiCl in RFV did not affect body weight, smell, or motor function significantly in either WT or 5XFAD mice.

**Conclusion:**

Intranasal LiCl in RFV ameliorates memory loss and depressive behavior in 5XFAD mice without side effects or organ toxicity, with chronic use. Intranasal LiCl in RFV effectively inhibited, oxidative stress, inflammation and pyroptosis in aged 5XFAD brains.

